# Assessing the toxicity of thiamethoxam, in natural LUFA 2.2 soil, through three generations of *Folsomia candida*

**DOI:** 10.1007/s10646-018-1922-8

**Published:** 2018-04-03

**Authors:** Cláudia de Lima e Silva, Justine Mariette, Rudo A. Verweij, Cornelis A. M. van Gestel

**Affiliations:** Department of Ecological Science, Faculty of Science, Vrije Universiteit, De Boelelaan 1085, Amsterdam, 1081 HV The Netherlands

**Keywords:** Multigeneration, Thiamethoxam, Actara®, Neonicotinoid, Springtail, Natural soil

## Abstract

In the field, long-term exposure is a rule rather than an exception. As a consequence, the relatively short-term standard toxicity tests may not be adequate for assessing long-term effects of pesticide exposure. This study determined the toxicity of the neonicotinoid thiamethoxam, both pure and in the formulation Actara® (25% active substance), to the springtail *Folsomia candida*, over three generations (P, F1 and F2). For the parental generation (P), the toxicity of pure thiamethoxam and Actara® did not differ significantly, with LC_50_s and EC_50_s of 0.32–0.35 and 0.23–0.25 mg a.s./kg dry soil, respectively. For the F1 and F2 generations, LC_50_s were >0.37 mg a.s./kg dry soil for both compounds. Actara was more toxic towards reproduction in the F1 generation (EC_50_ 0.16 mg a.s./kg dry soil) than pure thiamethoxam (EC_50_ 0.23 mg a.s./kg dry soil). For generation F2, there was no significant difference in the toxicity of the compounds towards reproduction, with EC_50_s of >0.37 and 0.30 mg a.s./kg dry soil for Actara® and pure thiamethoxam respectively. These results suggest a slight decrease in the toxicity of the compounds throughout the generations tested. The similarity in the toxicity of pure and formulated thiamethoxam indicates that the ingredients in the formulation Actara® do not enhance toxicity.

## Introduction

Neonicotinoids are neurotoxic insecticides, introduced in the market in the beginning of the 1990s with the release of imidacloprid. They are divided into three chemical classes: N-nitroguanidines (imidacloprid, thiamethoxam, clothianidin and dinetofuran), Nitromethylenes (nitenpyram) and N-cyanoamidines (acetamiprid and thiacloprid). Since their release there has been an increase in the use of these plant protection products worldwide, due to their broad-spectrum insecticidal activity, efficacy in the mode of action which translates into lower dosages applied in the field, and different forms of application, such as seed dressing (Jeschke and Nauen 2008; Jeschke et al. 2011). Despite the advantages of these compounds, residues may stay in the soil potentially threatening non-target organisms, including soil invertebrates (Goulson 2013; Dittbrenner et al. 2011, 2012; Pisa et al. 2015, 2017; van der Sluijs et al. 2015; Bonmatin et al. 2015, van Gestel et al. 2017). In 2013, the European Commission requested technical assistance of the European Food Safety Agency (EFSA) in order to review new evidence, published in 2012, concerning the toxicity of neonicotinoids towards bees. The Commission decided to restrict the use of imidacloprid, thiamethoxam and clothianidin for seed dressing of crops attractive to bees, with some exceptions (EU 2013). These compounds can still be used as plant protection for different types of crops.

The half-life of some neonicotinoids in soil is >100 days and for thiamethoxam it can reach 355 days (Bonmatin et al. 2015), indicating that the concentration of the residues in soil might increase due to repeated application in the field, leading to unknown long-term effects in soil invertebrates. Tennekes and Sánchez-Bayo (2013) suggested a delayed toxicity of imidacloprid and thiamethoxam, with cumulative effects over time of exposure pointing towards the need for long-time exposure tests for assessing the full impact of these compounds.

A *Folsomia candida* toxicity test, for instance, lasts 28 days (OECD 2009), covering part of the juvenile and adult stage and 1–2 weeks of the life of the offspring. In agricultural fields, however, thiamethoxam can be applied more than once within a year, leading to the accumulation of residues in soil. This may eventually cause a drastic reduction of soil invertebrate populations.

The aim of this study was to assess the toxicity of the neonicotinoid thiamethoxam, pure and in the formulation Actara®, in freshly spiked LUFA 2.2 soil, throughout three generations of the springtail *Folsomia candida*, in order to test two hypotheses: 1. Actara® is more toxic than pure thiamethoxam due to the presence of additives and other ingredients used in the formulation; 2. The toxicity of thiamethoxam, pure or in the formulation Actara®, will change throughout the generations, due to metabolic or soil transformation of the compounds into metabolites e.g. clothianidin (Nauen et al. 2003), that can present the same toxicity as the parent compound.

## Materials and methods

### Test soil and treatments

All tests were performed using natural standard LUFA 2.2 soil (LUFA Speyer, Germany), having approximately 1.6% organic carbon, water holding capacity (WHC) of 45%, and soil pH (0.01 M CaCl_2_) measured in a preliminary test, ranging between 5.03 and 5.87. The neonicotinoid thiamethoxam (99.6% a.s.; Fluka Sigma Aldrich; Germany) is a six-membered ring system, with a density of 1.57 g/ml at 20°C, presented as a slightly creamy and crystalline powder, water solubility at 20°C of 4100 mg/l, and octanol-water partition coefficient at pH 7, 20°C of 0.741 (Log K_OW_ −0.13) (Jeschke et al. 2011). Thiamethoxam formulation Actara® (250 W.G. a.s.) is a liquid product, with a density of 1.11–1.15 g/cm^3^ at 20°C, and is used against insect pests in a number of vegetables and citrus. In these crops, Actara® is applied as a foliar spray at rates of 93–345 g/ha and 207–345 g/ha, respectively (Syngenta 2009).

Stock solutions of both compounds were prepared in milli-Q water in order to spike the chemicals in the test soil and at the same time bring its moisture content to 50% of the water holding capacity (WHC). The concentration range used for both compounds was: Control −0.04 −0.12 −0.37 −1.1 −3.3 −10 mg a.s./kg dry soil. Due to the toxic effect of the neonicotinoids, the three highest concentrations had to be dropped for F1 and F2 generations. Tests were performed in 100 ml glass jars, containing 30 g moist soil, using five replicate test jars with animals, and two replicates containing approximately 15 g of soil each, without animals (soil analysis). All the test jars were weighed at the start of the test, so the water loss could be checked and replenished, if needed, on a weekly basis.

### Test Organism

*Folsomia candida* Willem 1902 is a cosmopolitan arthropod, belonging to the Collembola order, which is phylogenetically related to the Insecta. The family Isotomidae have a ventral tube or collophore which is involved in the exchange of fluids with the external environment, being an important route of exposure to contaminants present in the pore water. Sexual maturity of *F. candida* is achieved between 21 to 24 days after hatching (sixth instar), and each individual can lay about 30 to 50 eggs per batch (Fountain and Hopkin 2005). Adults of this species were taken from cultures at the Department of Ecological Science at the Vrije Universiteit in Amsterdam. In order to obtain synchronized animals, adults were transferred from the culture to 125 ml translucent plastic boxes with a 2 cm layer of plaster of Paris and activated charcoal (8:1), moistened with water, and left for a period of 2–3 days to lay eggs, after which they were removed. The eggs were incubated under a 12 h light/12 h dark regime at 20°C and 75% relative humidity, where they hatched. Juveniles of 10–12 days old were used for starting the test for the parental (P) generation.

### Experimental set-up

Three generations of *F. candida*, P, F1 and F2, were assessed for the toxicity of thiamethoxam and its formulation, Actara®. Ten age-synchronized animals (10–12 days old) were added to the replicate test jars in generation P. For generations F1 and F2 the age of the animals transferred was around 8–9 days. The tests for both compounds with the parental generation started on the same day, but due to time constraints the test with Actara® was finished after 27 days of exposure and that with the pure compound after 28 days. The same procedure was performed for ending the tests with the F1 (after 35–36 days) and F2 (28 days) generations. At the end of the tests, the jars were flooded with water to allow the animals to float and being photographed using a Nikon COOLPIX P510 camera. The images were analysed using the software ImageJ, a Java-based processing program adjusted for counting the animals. For the P and F1 generations, only the juveniles produced in all replicates of each concentration were transferred, using a sieve, to a single container with a plaster of Paris bottom, and left overnight in a climate room, at 20 ± 2 °C, with a 75% relative humidity and a photoperiod of 16:8 h dark:light. On the following day, batches of 10 randomly selected animals were transferred to jars, with their respective concentration, filled with soil freshly spiked, and covered with an opaque black plastic lid, loosely attached to the rim to allow for aeration. At the start and every week a few grains of dry baker’s yeast (Instant yeast from Algist Bruggeman N.V, Ghent, Belgium) and water were added to the test vessels if needed.

### Chemical analysis

Soil samples of both tests with thiamethoxam and Actara® were taken on the first day (*t* = 0) for each generation and on the last day of the test (*t* = 28) only for the parental generation. The samples were analysed for thiamethoxam by the commercial certified analytical laboratory Groen Agro Control in Delfgauw, The Netherlands, using LC-MSMS. Detection limit was 0.01 mg/kg dry soil.

### Data analysis

Using data on the measured concentrations of thiamethoxam, half-life (DT_50_) values were estimated assuming first order degradation kinetics. The equation C = C_0_ × e^−*k*×*t*^ was fitted to the data, with C = concentration at time *t* in days, C_0_ is concentration at *t* = 0 and *k* = degradation rate constant (day^−1^). DT_50_ was derived as ln(2)/*k*.

All the dose-response data was analysed using the package “drc”(dose response curve) in the software R, using a general model fitting function–drm, with the option for model select (Ritz and Streibig 2005). The endpoints LC_50_, addressing the effects on adult survival, and EC_x_, addressing sub-lethal effects on the juvenile population were expressed on the basis of nominal soil concentrations.

## Results

Concentrations of thiamethoxam spiked in the soil on the first day of the experiment (*t* = 0) were within 75–100% of the nominal ones. The recovery rate was slightly lower for pure thiamethoxam than for Actara® but degradation seemed to be faster for the latter (Table 1). Estimated DT50 values showed a dose-related decrease (from 156 to 112 days; average 139 days) for pure thiamethoxam and a dose-related increase for Actara® (from 46 to 134 days; average 84 days) (Table 1).

**Table 1:** Nominal and measured concentrations (mg/kg dry soil) of thiamethoxam in LUFA 2.2 soil at the start (*t* = 0) and end (*t* = 28 days) of the first (parental) generation test with *Folsomia candida* exposed to pure thiamethoxam or the formulation Actara®

Thiamethoxam	Nominal Concentration (mg/kg dry soil)	Measured Concentration (mg/kg dry soil)	Recovery Rate (%)	Nominal Concentration (mg/kg dry soil)	Measured Concentration (mg/kg dry soil)	Recovery Rate (%)	DT50 (days)
PURE	*t* ***=*** *0*			*t* ***=*** *28*			
	0	<0.01		0	<0.01		
	0.12	0.09	75	0.12	0.08	66	165
	0.37	0.31	84	0.37	0.27	65	140
	1.1	0.94	85	1.1	0.79	72	112
ACTARA^®^	*t* ***=*** *0*			*t* ***=*** 27*			
	0	<0.01		0	<0.01		
	0.12	0.12	100	0.12	0.08	67	46
	0.37	0.35	95	0.37	0.27	73	72
	1.1	1.0	90	1.1	0.87	79	134

*due to time constraints the test with Actara® had to be finished one day earlier

Tests with generations F1 and F2 did not meet the validity criteria (OECD 2009) for control survival, with 62–70 and 50% survival, respectively (>80% survival) (Table 2). The coefficient of variation for the number of juveniles produced by the controls of the F1 generation in the test with pure thiamethoxam was 60%, while it was 51% for the controls of the test on Actara® with generation F2.

**Table 2:** Validity criteria and control performance of *Folsomia candida* exposed for the three generations to thiamethoxam pure or in the formulation Actara®

Generation	Thiamethoxam	Mean adult mortality (%)	Mean number of juveniles	Coefficient of variation (%)
P	Pure	6	970	10
	Actara®	10	737	12
F1	Pure	30	548	60
	Actara	38	775	12
F2	Pure	50	475	24
	Actara®	50	264	51
Validity criteria		<20%	>100	<30%

**Fig. 1:**
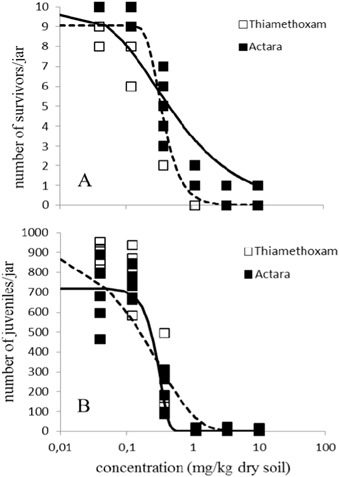
Dose-response relationships for the effect of thiamethoxam, pure and in the formulation Actara®, on the parental generation of *Folsomia candida* after 28 days exposure in LUFA 2.2 soil. A: Effects on survival (LC_50_ 0.32–0.35 mg a.s./kg dry soil); B: Effects on reproduction (EC_50_ 0.23–0.25 mg a.s./kg dry soil)_._ Concentrations are nominal values at the start of the test. Points are measured values, lines show the fit of a dose-response model to the data. Dashed lines are for Thiamethoxam, solid lines for Actara

**Fig. 2:**
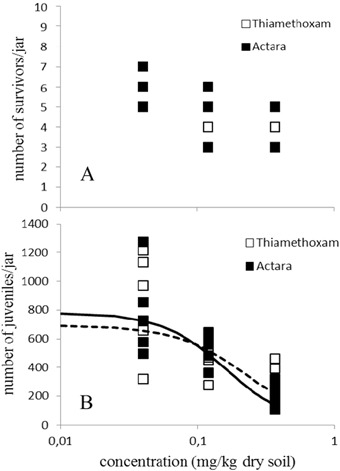
Dose-response relationships for the effect of thiamethoxam, pure and in the formulation Actara®, on generation F1 of *Folsomia candida* after 35 days exposure in LUFA 2.2 soil. A: Effects on survival (LC_50_ > 0.37 mg a.s./kg dry soil); B: Effects on reproduction (EC_50_ pure thiamethoxam 0.23–0.25 mg a.s./kg dry soil; EC_50_ Actara® 0.16 mg a.s./kg dry soil). Concentrations are nominal values at the start of the test. Points are measured values, lines show the fit of a dose-response model to the data. No dose-response curve could be generated for effects on springtail survival of both compounds. Dashed line is for thiamethoxam, solid line for Actara®

**Fig. 3:**
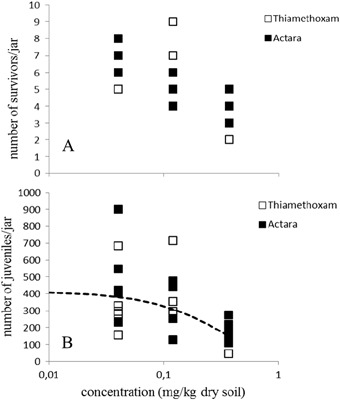
Dose-response relationships for the effect of thiamethoxam, pure and in the formulation Actara®, on generation F2 of *Folsomia candida* after 28 days exposure in LUFA 2.2 soil. A: Effects on survival (LC_50_ > 0.37 mg a.s./kg dry soil); B: Effects on reproduction (EC_50_ pure thiamethoxam 0.30 mg a.s./kg dry soil; EC_50_ Actara® > 0.37 mg a.s./kg dry soil). Concentrations are nominal values at the start of the test. Points are measured values, line shows the fit of a dose-response model to the data. No dose-response curve could be generated for the effects on springtail survival of both compounds and reproduction effect of Actara®

Steep dose-response curves were found for adult survival of the P generation (Fig. 1), with LC_50_s of 0.32 and 0.35 mg a.s./kg dry soil, for pure thiamethoxam and Actara® respectively (Table 3), with no significant difference between these values (*p* > 0.05). Above these concentrations, not enough juveniles were produced in order to be transferred to the next generation (F1). The maximum concentration tested (0.37 mg a.s./kg dry soil) did not kill 50% of the adult population in generations F1 and F2, and as a consequence a dose response curve could not be produced (Figs. 2 and 3). Reproduction of the P generation seemed directly influenced by the reduction in the number of adults (Fig. 1), with EC_50_s of 0.23 and 0.25 mg a.s./kg dry soil, which did not differ significantly (*p* > 0.05). The EC_50_s for the toxicity of pure thiamethoxam and Actara® to the reproduction of the F1 generation also did not differ significantly (Fig. 2), as can be seen from the overlapping confidence intervals (Table 3). For the F2 generation, there seemed to be a slight reduction in the sensitivity of the animals towards both endpoints (Fig. 3), with LC_50_s > 0.37 mg a.s./kg dry soil and EC_50_s of 0.30 and > 0.37 mg a.s./kg dry soil, for pure thiamethoxam and Actara®, respectively.

**Table 3:** Endpoints analysed for the effects of thiamethoxam, pure and in the formulation Actara® on the survival and reproduction of *Folsomia candida* exposed in LUFA 2.2 soil for three generations

	Parental generation		F1 generation		F2 generation	
Endpoints (mg a.s./kg dry soil)	Thiamethoxam Pure	Actara®	Thiamethoxam Pure	Actara®	Thiamethoxam Pure	Actara®
LC_50_	0.32 (0.30–0.40)	0.35 (0.30–0.40)	>0.37	>0.37	>0.37	>0.37
EC_50_	0.23 (0.20–0.30)	0.25 (0.16–0.34)	0.23 (0–0.52)	0.16 (0.09–0.20)	0.30 (0.09–0.50)	>0.37
EC_20_	0.12 (0.070–0.20)	0.18 (0.06–0.30)	0.09 (0–0.20)	0.08 (0.03–0.10)	0.20 (0–0.63)	0.25 (0–1.1)
EC_10_	0.07 (0.03–0.10)	0.16 (0.03–0.30)	0.06 (0–0.14)	0.06 (0.01–0.11)	0.15 (0–0.64)	0.20 (0–1.3)

## Discussion

The validity criteria for adult mortality and the coefficient of variation (OECD 2009) were met only for generation P tests. Mean adult mortality for generations F1 and F2 did exceed the criterion of 20% set by (OECD 2009), while the coefficient of variation for juvenile numbers exceeded the limit of 30% only for the controls of the tests with pure thiamethoxam on generation F1 and Actara® on generation F2 (Table 2). One of the factors that might have contributed to the lower control survival and higher variation in control reproduction is the age of the juveniles when transferred, which was 8–9 days (F1 and F2) instead of the aimed 10–12 days. The difference in the age of the animals that were introduced in the tests for generations P and F1 seemed to affect adult survival, but did not affect reproduction. According to Crouau and Cazes (2003), there is no significant difference in the reproduction of animals aged 10 or 11 days old, but older animals do produce more offspring than younger ones.

If the duration of the exposure of generation F1 was kept at 28 days, and assuming that the life cycle of *F. candida* was not affected by living in LUFA 2.2 soil, the age of the juveniles at the end of the test period would be around 2–4 days. To compensate for this, the duration of the exposure for generation F1 was extended to 35 days, as suggested by Crouau and Cazes (2003), Leon-Paumen et al. (2008) and Ernst et al. (2016). The increased control mortality might also be due to the stress of handling, that involved sieving, transfer of the animals to plaster, overnight incubation, handling again and transfer to LUFA 2.2 soil. Another aspect that should be considered is the possible heterogeneity in age and size of the animals derived from different clutches produced (Campiche et al. 2007; Ernst et al. 2016).

Despite these factors, the numbers of juveniles produced was at least twice the minimum of 100 animals required for all the generations, regardless the compound (Table 2). This suggests that the animals were fit, and the low survival did not impact their reproduction.

The toxicity of both pure thiamethoxam and the formulation Actara® towards survival throughout the generations remained mostly stable, with a slight decrease from generation P onwards. Toxicity towards reproduction was slightly increased for Actara® on the F1 generation, but overall it did not present a significant difference between the pure compound and its formulation (Table 3). This indicates that that the toxicity of Actara® is dominated by the active substance and not enhanced by the formulating agents.

There is a gap in knowledge on the toxicity of thiamethoxam to non-target soil invertebrates. There is also some variation in the existing data, which could be related to differences in the types of soil used or to adjustments on the methodology (Table 4). Alves et al. (2014) tested a different formulation of thiamethoxam (Cruiser FS® 35% a.s) in chronic and acute assays with *F. candida* in a Tropical Artificial Soil (TAS) and found low toxicity with LC_50_ > 1000 mg a.s./kg dry soil. Our study, however, showed a higher toxicity of thiamethoxam, with LC_50_s of 0.32–0.35 mg a.s./kg dry soil. This discrepancy might be related to the different methodology applied (14 days exposure for LC_50_ test) and the use of a different soil type with different contents and types of organic matter. Imidacloprid binds strongly to organic matter (Cox et al. 1997), and therefore is less bioavailable at higher organic matter content. Since thiamethoxam and imidacloprid belong to the same chemical family, it is assumed that they behave similarly (Cox et al. 1997). Other points to be considered are the test conditions and their impacts on the animals. Alves et al. (2014) exposed European cultures of *F. candida* at temperatures of 23 ± 2°C, with a photoperiod of 12:12 h light:dark in order to mimic tropical conditions. The optimum temperature for culturing this species, however, is 20°C, and for success in hatching it is 21°C (Fountain and Hopkin 2005). All these factors might have contributed to the large discrepancy in LC_50_ values. Tests performed using the same type of soil, same methodology and same exposure conditions do provide similar results. E.g. exposures of *F. candida* to imidacloprid in LUFA 2.2 soil performed under the same laboratory conditions presented similar LC_50_ and EC_50_ values (van Gestel et al. 2017, de Lima e Silva et al. 2017), confirming replicability of the test (Table 4).

**Table 4:** Literature review on the toxicity of imidacloprid and thiamethoxam (both pure and formulation) to the survival and reproduction of *Folsomia candida*

Neonicotinoid	LC_50_ (mg a.s./kg)	EC_50_ (mg a.s./kg)	Type of soil	Reference
Gaucho FS® (60% a.s.)–Imidacloprid	20.96^a^	(*)	TAS^#^ (fine sand > 50%; kaolinite clay and powdered coconut husk (7:2:1)	Alves et al. 2014
Cruiser FS® (35% a.s.)– Thiamethoxam	>1.000^a^	(*)	TAS (fine sand > 50%; kaolinite clay and powdered coconut husk (7:2:1)	Alves et al. 2014
Confidor WS® (75% a.s.)–Imidacloprid	0.11	0.15	ISO (sphagnunm peat, kaolin clay, quartz sand)	Idinger 2002
Imidacloprid (99% a.s.)	0.86	0.26	sphagnum peat (10%), kaolinite clay (20%), quartz sand (70%)	Reynolds 2008
Imidacloprid (99% a.s.)	0.44	0.29	Lufa 2.2 soil (1.5% organic carbon, pH in 0.01 M CaCl_2,_ WHC of 43.5% of its dry weight)	van Gestel et al. 2017
Imidacloprid (99% a.s.)	0.47	0.26	Lufa 2.2 soil (1.5% organic carbon, pH in 0.01 M CaCl_2,_ WHC of 43.5% of its dry weight)	de Lima e Silva et al. 2017

*not available

^a^14 days exposure; ^#^ Tropical Artificial Soil

Thiamethoxam was as toxic as imidacloprid for survival and reproduction when comparing the LC_50_s and EC_50_s of 0.32–0.35 and 0.23–0.25 mg a.s./kg dry soil obtained for the parental generation with the values of 0.44–0.47 and 0.26–0.29 mg a.s./kg dry soil for imidacloprid (De Lima e Silva et al. 2017; van Gestel et al. 2017). This was expected since both compounds belong to the same chemical family–N-nitroguanidines. Neonicotinoids act upon the nervous system of insects, by disrupting the stimulus, preventing depolarization, leading to paralysis or cell energy exhaustion (Thany 2011). Although springtails do not belong to the Insecta, they are phylogenetically related and their nervous system therefore might be affected by the neonicotinoids acting on similar receptor sites. Thiamethoxam has some affinity to the binding sites of imidacloprid in the nAChRs of the insects, which partly explains its toxicity, but the compound also binds to specific sites (Thany 2011). Although it was supposed that thiamethoxam would have affinity to the binding sites of clothianidin, since it can be metabolised in plants and insects into clothianidin (Nauen et al. 2003, Simon-Delso et al. 2015), these compounds do bind to different sites or nAChR subtypes (Wellmann et al. 2004; Wiesner and Kayser 2000; Kagabu et al. 2005). According to Thany (2011), clothianidin induces a monophasic depolarization which cannot be reversed, while the depolarization caused by thiamethoxam can be reversed after wash-out. This different behaviour towards the nAChRs indicates that even if clothianidin was formed, in soil or through the metabolism of the animals, its influence on the toxicity of thiamethoxam in this test would be minimum, since the sensitivity of the animals decreased throughout the test. This indicates some recovery, which would not be possible with the irreversible binding of clothianidin.

Based on the application rates of Actara® provided by the producer (Syngenta 2009), and considering that half the dose would reach the soil and become homogenously distributed in the top 5 cm soil layer, the predicted environmental concentration (PEC) was estimated to be 0.063–0.23 mg/kg dry soil for vegetables and 0.14–0.23 mg/kg dry soil for citrus (foliar spray). When comparing these PEC values with the EC_50_ values for the toxicity of thiamethoxam (Table 3), reproduction of *F. candida* seems to be at risk already with only one application per year. The application guide (Syngenta 2009) indicates that the product has to be applied with an interval of 5–7 days between applications. According to Goulson (2013), the half-life of thiamethoxam pure in soil may range between 7 and 353 days. In our study (Table 1), thiamethoxam showed to be fairly stable, with DT50 values of 46–134 days for the Actara® treatments and 112–165 days for the treatments with the pure compound. Therefore the application interval suggested by the producer might lead to thiamethoxam accumulation in soil to concentrations potentially reaching the LC_50_, putting the population of *F. candida* in agricultural fields at risk of extinction.

Standard tests to assess the toxicity of compounds to *F. candida* (OECD 2009; ISO 1999) use exposure times of 28 days, and focus on just a part of its life cycle (last phase of juvenile period, first reproductive phase, egg and early juvenile phase). Although covering some important and possibly sensitive parts of the life cycle, this reduces the possibility to evaluate the potential risks (sub-lethal effects) for future generations exposed to the compound. Multigeneration tests are more suitable for assessing sub-lethal chemical effects, and the potential for recovery of the population. Van Gestel et al. (2017) showed a potential for recovery of *F. candida* populations after three generations exposed to thiacloprid. In this case, the recovery was connected with the half-life of the compound (10–12 days), resulting in a reduced exposure of subsequent generations that were in contact to soil spiked only once at the start of the experiment. In our study, thiamethoxam was freshly spiked into the test soil at the start of each generation, so to ensure a more constant exposure level. Nevertheless, the toxicity of the compound through generations presented a slight reduction, which might suggest adaptation or recovery of the population. The latter could be associated with the reversible binding of thiamethoxam to the subunits and nAChRs in the nervous system, and the potential for detoxification of *F. candida*.

Different designs can be applied for a multigeneration test, depending on the questions to be answered: spiking the soil only once, freshly spiking the soil for each new generation to ensure more or less continued exposure, transfer to clean soil in order to allow decontamination followed by exposure, and so on (Campiche et al. 2007; Ernst et al. 2016; van Gestel et al. 2017). In this study, we decided to freshly spike the test soil for each generation, in order to simulate continuous exposure due to repeated applications in the field. We also used an extended exposure time for generation F1, in order to allow the juveniles to develop further before being transferred. Although the latter did not decrease adult mortality, the multigeneration assessment proved to be suitable to determine the toxicity of thiamethoxam. It did not only show that the thiamethoxam is more toxic than imidacloprid (for parental generation), but results also suggest there is a potential for the population to recover throughout the generations. The latter does however, need confirmation with a more in-depth analysis of long-term effects combined with an analysis of biochemical and molecular aspects of thiamethoxam uptake, metabolism and intoxication in *F. candida*.

## Conclusions

There was no significant difference in the toxicity between pure thiamethoxam and its formulation Actara®, indicating that other ingredients and additives present in the formulation did not interfere with the toxicity of the compound. The toxicity of both compounds to survival and reproduction did not increase throughout the generations as hypothesised, instead it showed a slight reduction. Although we did not check for the presence of clothianidin in soil or test organisms, it is likely this metabolite did not contribute to the toxicity of thiamethoxam as this would have increased rather than decreased its toxicity. The use of a multigeneration test, with the extension of the duration of the exposure for generation F1, proved to be suitable for assessing the sub-lethal effects of thiamethoxam. Thiamethoxam proved to be as toxic as imidacloprid to the survival and reproduction of *Folsomia candida*, presenting a risk to springtail in agricultural fields where these compounds are used.
